# Virulence Gene Profile, Antimicrobial Resistance and Multilocus Sequence Typing of *Salmonella enterica* Subsp. *enterica* Serovar Enteritidis from Chickens and Chicken Products

**DOI:** 10.3390/ani12010097

**Published:** 2022-01-01

**Authors:** Zunita Zakaria, Latiffah Hassan, Zawiyah Sharif, Norazah Ahmad, Rohaya Mohd Ali, Suraya Amir Husin, Norfitriah Mohamed Sohaimi, Shafini Abu Bakar, Bashiru Garba

**Affiliations:** 1 Institute of Bioscience, Universiti Putra Malaysia, Serdang 43400, Malaysia; 2 Department of Veterinary Pathology and Microbiology, Faculty of Veterinary Medicine, Universiti Putra Malaysia, Serdang 43400, Malaysia; garba.bashiru@udusok.edu.ng; 3 Department of Veterinary Laboratory Diagnostics, Faculty of Veterinary Medicine, Universiti Putra Malaysia, Serdang 43400, Malaysia; latiffah@upm.edu.my (L.H.); fitriahsohaimi@upm.edu.my (N.M.S.); 4 Food Safety and Quality Division, Ministry of Health, Putrajaya 62675, Malaysia; zawiyahsharif@moh.gov.my (Z.S.); shafiniabubakar@moh.gov.my (S.A.B.); 5 Veterinary Public Health Division, Department of Veterinary Services Malaysia, Putrajaya 62630, Malaysia; norazah@imr.gov.my (N.A.); rohaya@dvs.gov.my (R.M.A.); 6 Medical Development Division, Ministry of Health, Putrajaya 62590, Malaysia; drsurayaamirhusin@gmail.com; 7 Faculty of Veterinary Medicine, Usmanu Danfodiyo University, Sultan Abubakar Road, City Campus Complex, Sokoto 840212, Nigeria

**Keywords:** *Salmonella* Enteritidis, chicken, multilocus sequence typing, antimicrobial resistance, virulence

## Abstract

**Simple Summary:**

We investigated the pathogenicity, antimicrobial resistance and multilocus sequence types of *Salmonella enterica* subspecies *enterica* serovar Enteritidis isolated from fresh chicken meat, ready-to-eat chicken meat as well as from cloacal swabs of live chickens in some selected locations within the central region of peninsular Malaysia. After culture and serotype confirmation of the *Salmonella* isolates, the genomic DNA was extracted and whole-genome sequencing was conducted using the NextSeq 550 System (Illumina, San Diego, CA, USA). In silico serotypes were determined with the aid of SeqSero WGS in silico software version 2, while multilocus sequence types, as well as virulence and antimicrobial resistance determinants, were all determined using the BioEasy Epinod pipeline. The *S*. Enteritidis isolates were found to harbour several virulence genes, with multidrug-resistance characteristics. The results of this investigation indicate the potential risks both humans and livestock are exposed to due to this foodborne pathogen.

**Abstract:**

This study was undertaken to determine the virulence, antimicrobial resistance and molecular subtypes of *Salmonella* in the Central Region of Peninsular Malaysia. A total of 45 *Salmonella* Enteritidis were detected from live chicken (cloacal swab), and chicken products (fresh and ready-to-eat meat) samples upon cultural isolation and serotyping. Similarly, an antimicrobial susceptibility test based on the Kirby Bauer disk diffusion method as well as antimicrobial resistance AMR genes, virulence determinants and multilocus sequence typing (MLST) typing were conducted after the Whole Genome Sequencing and analysis of the isolates. The results indicate that sequence types ST1925 (63.7%), and ST11 (26.5%) were the predominant out of the seven sequence types identified (ST292, ST329, ST365, ST423 and ST2132). The phenotypic antimicrobial profile corresponds to the genotypic characterization in that the majority of the isolates that exhibited tetracycline, gentamycin and aminoglycoside resistance; they also possessed the *tetC* and *blaTEM* β-Lactam resistance genes. However, isolates from cloacal swabs showed the highest number of resistance genes compared to the chicken products (fresh and ready-to-eat meat) samples. Furthermore, most of the virulence genes were found to cluster in the *Salmonella* pathogenicity island (SPI). In this study, all the isolates were found to possess SPI-1, which codes for the type III secretion system, which functions as actin-binding proteins (SptP and SopE). The virulence plasmid (VP) genes (*spvB*, *spvC*) were present in all genotypes except ST365. The findings of this study, particularly with regard to the molecular subtypes and AMR profiles of the *Salmonella* Enteritidis serotype shows multidrug-resistance features as well as genetic characteristics indicative of high pathogenicity.

## 1. Introduction

The Malaysian Action Plan on Antimicrobial Resistance (MyAp-AMR 2017–2021) was established to aggressively address the threat posed by the emergence of antimicrobial resistance among pathogens in the country. Among their activities was to study strains from human infections, food-producing animals, and raw retail meats and aquaculture [1]. This is a follow-up to the already functional National Surveillance of Antibiotic Resistance (NSAR) program which has been active in Malaysia since 1988 [2]. The MyAp-AMR program is aimed at controlling the emergence and further spread of AMR, by educating the relevant stakeholders in the health industry particularly the healthcare administrators, and the livestock and aquaculture subsector on the dangers and public health importance of AMR [1]. Among the priority pathogens are *Salmonella*, *Campylobacter*, *Klebsiella*, *Staphylococcus*, *Escherichia coli* and *Streptococcus*, among a host of other important foodborne pathogens [1]. The program integrated the National Surveillance and a “One Health” approach to determine the magnitude of antimicrobial resistance in bacteria spreading through the food chain that eventually result in diseases in humans and loss in productivity in livestock.

*Salmonella enterica subspecies enterica* serovar Enteritidis is a serotype of global public health significance [3]. It is one of the most common strain responsible for human infection worldwide second to *S*. Typhimurium [4]. Infections are usually characterized by mild self-limiting gastroenteric manifestations. However, in severe cases the bacteria can cause meningoencephalitis, septic arthritis and other extraintestinal illnesses in infants and immunocompromised adults [5]. In resource-poor countries, *S*. Enteritidis is an important cause of morbidity and mortality in children. It is also frequently incriminated in foodborne outbreaks [6]. In recent years, the world has been witnessing the emergence of invasive nontyphoidal *Salmonella* lineages with an extended multidrug-resistance range [7,8,9]. The emergence, although variable, represents a major public health concern, especially in Asia and Sub-Saharan Africa [10,11,12].

Molecular typing of *Salmonella* has proven to be an important infection control tool as it helps in monitoring the prevalence of specific strains in a cluster of unrelated outbreaks or within human healthcare institutions. It also provide information on the genetic relatedness of strains that are useful for surveillance as well as during an epidemiological investigation of outbreaks [13]. Two of the most popular molecular typing methods used in *Salmonella* are the Pulse Field Gel Electrophoresis (PFGE) and the Multilocus Sequence Typing (MLST). Although both techniques are robust and reliable, PFGE requires rigorous standardization protocols; while MLST can be used to generate unambiguous data from different laboratories and can be used to study evolutionary relationships between isolates across the globe [14,15]. Whole-genome MLST on the other hand can be used to generate isolate-specific genetic fingerprints suitable for assessing epidemiological relatedness based on single nucleotide polymorphisms (SNPs), as well as insertions and deletions [15].

The isolation of *Salmonella* and other foodborne pathogens from human clinical samples, animals and food of animal origin have been consistently conducted and its trends monitored by the Malaysian Ministry of Health in collaboration with the Department of Veterinary Service, Malaysia. Recent reports by the National Surveillance of Antimicrobial Resistance NSAR indicate that there has been an increase in the antibiotic resistance profile of *Salmonella* spp., with multiple resistance (multidrug-resistance—MDR) to ciprofloxacin (3.4%) and ampicillin (25%) in humans [16]. Similarly, investigations conducted by the Department of Veterinary Services Malaysia (2013/2014) to determine the prevalence and antimicrobial patterns of *Salmonella* isolates from chicken meat within the Central region of Peninsular Malaysia revealed a high prevalence of *Salmonella* contamination with a variety of antimicrobial resistance profiles including MDR phenotypes among the *Salmonella* isolates [1].

This study was undertaken to determine the virulence and antimicrobial resistance profile of *Salmonella* Enteritidis isolated from chicken and chicken products in Malaysia. It also sought to determine the serotype diversity of the isolates using Whole Genome Sequencing data. The results will benefit the current prevention and control efforts of the Malaysian Government by strengthening the knowledge and evidence base concerning the status of *Salmonella* in the sampled locations, with particular emphasis on *S.* Enteritidis which is one of the most prevalent strains causing invasive salmonellosis globally.

## 2. Materials and Methods

### 2.1. Bacterial Isolates

The isolates used in this study were obtained from stock cultures from the Department of Veterinary Services (DVS) and Food Safety and Quality Division laboratories, Ministry of Health. They were isolated from faecal swabs, and fresh/ready-to-eat chicken meats (food) samples collected from April 2016 to November 2018, as part of the *Salmonella* collections for the National AMR surveillance program. The samples were obtained within the central region of Peninsular Malaysia that comprises the states of Selangor and Melaka. The faecal swabs were collected from poultry farms, while the chicken products (fresh meat samples and ready-to-eat chicken) were collected from retail stores and food vendors, respectively.

The 45 *Salmonella* isolates used in this study were obtained from ready-to-eat chicken meat at retail markets (*n* = 7), raw chicken meat (*n* = 11), and (*n* = 27) cloacal swabs. The procedure for the culture and isolation used in all the laboratories entails collecting sterile swabs, or 1 g of homogenized meat sample to be inoculated in 9 mL of buffered peptone water and incubated at 37 °C for 18–24 h [17]. Subsequently, 100 µL of the pre-enriched, buffered peptone water was transferred into another 10 mL of Rappaport-Vassiliadis (RVS) broth (Oxoid, Basingstoke, UK) and incubated at 41 °C for 24 ± 2 h for selective enrichment. This was then followed by streaking of the RVS broth enrichment culture onto xylose lysine deoxycholate (XLD) agar (Oxoid, UK) and Brilliant Green agar (Oxoid, UK), and the plates were incubated for 24–48 h at 37 °C. The resultant presumptive *Salmonella* colonies grown on the XLD and Brilliant green agar (BGA) plates were then subcultured on nutrient agar plates for isolation of distinct colonies. Biochemical characterization (catalase, urease, SIM) and serotyping (slide agglutination with O and H antigen-specific sera) were performed at the District Laboratories under the jurisdiction of the Department of Veterinary Services, Malaysia, based on the OIE standard method. Additionally, *Escherichia coli* (ATCC 25922) was included in the study as a negative control.

### 2.2. Phenotypic Antimicrobial Susceptibility Testing

The *S.* Enteritidis positive isolates were subjected to an antimicrobial susceptibility test by the Kirby–Bauer disc diffusion method on Mueller Hinton agar (Merck, Darmstadt, Germany) using nine antibiotic discs (Oxoid LTD, UK): ampicillin (Amp), chloramphenicol (C), gentamicin (CN), streptomycin (S), sulfamethazine/trimethoprim (SXT), tetracycline (TE), ceftiofur (EFT), cefotaxime (CTX), and ciprofloxacin (CIP). These drugs are part of the Malaysia Ministry of Health Medicines Formulary (Formulari Ubat Kementerian Kesihatan Malaysia) and are among the drugs commonly used in healthcare clinics in Malaysia [18]. Results were interpreted following the Clinical and Laboratory Standards Institute guidelines (CLSI 2018, 4th Ed) [19] while *Escherichia coli* ATCC 25922 was used as control.

### 2.3. Whole Genome Sequencing

About 2 mL of an overnight culture of the *Salmonella* isolates were centrifuged at 5000× *g* for 10 min and the genomic DNA was extracted from the resultant pellet using QIAamp DNA Mini Kit (Qiagen). The Nextera™ DNA Flex Library Prep Kit was used for the preparation of the genomic libraries and the Whole Genome Sequencing was performed on the NextSeq 550 System (Illumina, San Diego, CA, USA). The good-quality sequencing reads were then assembled using SPAdes (SPAdes version 3.9.0) to obtain contigs [20]. All the assembled contig sequences from the 45 *Salmonella* isolates as well as *Escherichia coli* K12 as control were subjected to comparative studies using the EPInod pipeline developed by BioEasy Sdn Bhd.

### 2.4. In Silico Serotype Prediction Using SeqSero

The serotype of the isolates was determined with the aid of SeqSero WGS in silico software version 2. The software allows for the serotype prediction from raw reads and genome assemblies. The software uses a k-mer-based algorithm which allows for the rapid serotype prediction from WGS data, and is built with additional sequence markers for the identification of *Salmonella* species and subspecies.

### 2.5. Determination of Salmonella Virulence and Resistance Determinants

The genome virulence factor analysis against the major *Salmonella* virulence factors was performed via the BLAST program (BLAST 2.5.0+) against the virulence factor database (VFDB) with the aid of EPInod software (BioEasy, Sdn Bhd). The genome comparison analysis was streamlined via the Abricate program against ResFinder and Card (Abricate–Version 0.8). The assembled contig sequences were subjected to comparative analyses using the EPInod, which included the Multilocus Sequence Typing (MLST), and virulence factor (VF) detection. The presence of the *Salmonella* virulence determinants was investigated by targeting some major gene determinants: the cell invasion protein, transmembrane proteins, secretion-system effector proteins and the putative transcriptional regulator protein, and in addition, the immune evasion, host-colonization factor and the type 1 fimbria adherence determinant [21]. Similarly, virulence genes were identified by mapping the Illumina raw reads against chromosomal and plasmid virulence genes deposited in the Virulence Factor Database for *Salmonella* (VFDB).

### 2.6. Multilocus Sequence Typing—MLST

The Multilocus Sequence Typing and characterization was performed after PCR amplification and sequencing using the reported conventional primers targeting the seven housekeeping genes: *aroC*, *dnaN*, *hemD*, *hisD*, *purE*, *sucA*, and *thrA* [22]. The sequence type for each isolate was assigned based on the set of alleles derived from the seven loci. The goeBURST Minimum Spanning Tree expansion was also used to visualize the possible evolutionary relationships between isolates using the PHYLOViZ software v2.0 online software [23].

## 3. Results

### 3.1. Phenotypic Antimicrobial Resistance Profiles

Twenty-nine (62.3%) of the 45 *S.* Enteritidis isolates were found to be resistant to one or more antimicrobials tested, while sixteen (37.7%) were susceptible to all the antimicrobials tested. The percentage of resistance to each of the tested antimicrobial drugs is presented in Table 1. Samples that showed multidrug resistance against Amp, CN, TE and S were all obtained from cloacal swabs from poultry farms in Selangor. However, 54% of the isolates exhibiting tetracycline resistance were from cloacal samples from poultry farms in Melaka.

### 3.2. Phenotypic and WGS Serotype Prediction

The traditional Kauffman–White scheme for serotyping found that all 45 isolates belong to the *S.* Enteritidis serogroup based on the O and H antigens (Table 2). While the SeqSero2 (SeqSero2 v1.1.0) serotype prediction showed 89.9% concordance with the traditional method while being discordant on the serotypes of four isolates. The SeqSero2 identified the four isolates of *S.* Ohio (S72-cloacal swab), *S.* Weltevreden (S77-cloacal swab) and *S.* Kentucky (S81-cloacal swab), while S63 (cloacal swab) was found to have the antigenic formula (8: z4, z24) which corresponds to either *S.* Albany or *S.* Duesseldorf, because both of these share the same antigenic formula as detected by the SeqSero2 software. Furthermore, the WGS in silico analysis identified one unique isolate with antigenic formula I 4:b:-(S87-cloacal swab), which is not listed in the Kauffmann White Scheme.

### 3.3. Antimicrobial Resistance Genes and Virulence Factor Determinants

The Whole Genome Sequence data from the positive *Salmonella* isolates were examined for the possession of antimicrobial resistance genes and virulence factor determinants. Overall, the most common resistance genes detected were *pmr c*, *pmr e*, *pmr f* and *aac (6’)-Iy* (Table 3). Similarly, the *Salmonella* isolates were also screened for virulence genes. A total of 122 virulence-factor determinants were detected in this study using the WGS. Notable among the common *Salmonella* virulence genes detected are plasmid-encoded fimbriae chaperone protein *PefD*, type III secretion-system effector *SpvC*, phosphothreonine lyase *Spv*, resistance to complement killing *Rck*, type III secretion-system effector *SseK1*, SPI-2-encoded T3SS, as well as *InvA*, and *Spa*, among others.

### 3.4. Multilocus Sequence Typing of S. Enteritidis Isolates

**Figure 1:**
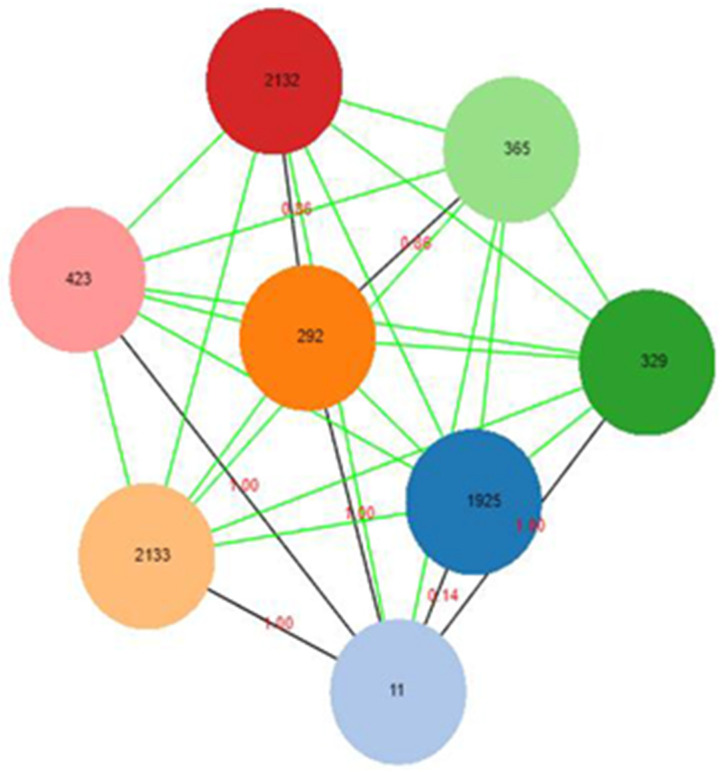
MLST Clonal Complex defined by goeBURST for *Salmonella* Enteritidis isolates. Each coloured node represents a clone in relation to their absolute distance, with ST11 being more closely related to ST1925.

The MLST analysis identified seven sequence types based on the MLST database results (Table 4). The most commonly detected allelic profiles were ST1925 (63%) and ST11 (26.5%). Other sequence types identified are ST292, ST329, ST365, ST423 and ST2132, which were observed in each of the three categories of samples studied (ready-to-eat chicken meat, fresh chicken meat, and cloacal swab). In the goEBURST dataset, lowering the level to seven results in detachment into five distinct groups with three singletons (STs 2132, 329 and 423), while STs 2132, 292 and 365 clustered together (Figure 1).

This implies that the connection between the detached nodes had at least seven differences. Similarly, the sample data set was used to generate a graph with an nLV (N Locus Variant) of level seven, which identifies sets of closely related nodes; and the results indicate that all the allele profiles with distances equal or below six were all connected.

## 4. Discussion

To date, serological typing of *Salmonella* remains the most popular tool for characterization during surveillance and epidemiological investigations [24]. However, molecular techniques are beginning to gain popularity as substitutes to the traditional Kauffman–White method. In this study, both the traditional and molecular (SeqSero2) methods were employed for the serotyping of the *Salmonella* isolates. The SeqSero2 was found to be a good alternative to the phenotypic serotyping, with 89.9% of the results in full agreement. This finding conforms with previous reports by Diep et al. and Ferrato et al. [24,25]. Both Diep et al. and Ferrato et al. used Check & Trace *Salmonella™*, a commercial DNA Microarray System to determine serotype designation of *Salmonella* isolates from clinical samples as well as the WGS-based serotype prediction tool (SeqSero) utilized in this study, and suggested that SeqSero is a very reliable replacement for the traditional serotyping method where WGS is implemented. The in silico method in this investigation showed improved performance over the traditional method by accurately predicting the antigenic formulae of the *Salmonella* serotypes in a manner consistent with the traditional phenotypic serotyping, albeit with some disparity. However, the claim for superiority for SeqSero2 can be affected by the fact that the same antigenic formula can be shared by strains from different subspecies, as observed with Albany and Dusseldorf (8: z4, z24) in this study. Similarly, SeqSero was not able to identify some *Salmonella* subspecies accurately because they differ only by minor epitopes which were often not available in the software [24]. Nevertheless, in comparison, this technique is much faster than the traditional phenotypic method, which usually requires a couple of days to complete or even longer when dealing with rare serotypes.

The emergence of multiple-drug-resistant, and highly pathogenic *S.* Enteritidis, signifies a serious threat to public health and food safety. Hence, in addition to identifying the serotypes, we sought to understand the genetic variability and virulence characteristics of the *Salmonella* isolates. This is because *S.* Enteritidis is a major zoonotic pathogen that constitutes a serious public health problem globally, especially with the rapid development and emergence of multidrug-resistant strains [5,26]. The strain is a common food contaminant, and has been incriminated in several outbreaks in Malaysia in recent years [11]. Moreover, human infections are being repeatedly reported, with chicken and chicken products being one of the most common sources of infection [11,27,28]. Therefore, it has become paramount to investigate and understand the potential role of chicken (meat and meat products) in the dissemination of *Salmonella* species.

The phenotypic AMR analysis showed that isolates from ready-to-eat meat samples (chicken meat products) were resistant to only tetracycline (5/7), while isolates from raw chicken meat and cloacal swab samples were resistant to multiple antimicrobials including tetracycline (4/11; 11/27), and ampicillin (0/11; 6/27) predominating. While varying levels of AMR resistance was observed among the isolates in both Selangor and Melaka, it is interesting to note that all the isolates that exhibited multidrug resistance were isolated from cloacal swabs samples collected from poultry farms in Selangor, with the majority of the isolates from Melaka showing resistance to tetracycline. This finding is similar to the findings of Ibrahim et al., where they investigated the prevalence of antimicrobial resistant *Salmonella* in the East Coast of the peninsular Malaysia [29]. *S.* Enteritidis isolates in Selangor have been reported to exhibit high resistance against several antimicrobials including penicillin, erythromycin and vancomycin [30,31,32,33]. However, in this study, resistance was observed against ampicillin, gentamicin, and tetracycline. Even though numerous potential vehicles of transmission for *Salmonella* are abound, commercial chicken meat has been identified as one of the most crucial food vehicles for these organisms [34]. The result from this study is therefore a further testament to the role of poultry in the spread of resistant *Salmonella* in Malaysia.

Animals and foods of animal origin, especially if prepared under poor hygienic conditions, can serve as a source of *Salmonella*. Therefore, it is not uncommon to observe a similar frequency of AMR from different sources, as observed in the present study. All the isolates from the ready-to-eat chicken meat in Selangor showed resistance to only tetracycline, except for one isolate that showed resistance to sulfamethazine/trimethoprim. Many studies have observed the rate at which foodborne pathogens are acquiring resistance to tetracycline and other commonly used antimicrobials in the food chain, which is a cause for concern. In the same vein, Zakaria et al. have also reported a high rate of tetracycline resistance among *Salmonella* isolated from poultry meat [8]. *S*. Enteritidis isolates have also been reported to show increasing resistance to the commonly used antimicrobials in livestock. In this study, the highest level of resistance was observed with tetracycline, followed by ampicillin. Although, we cannot say exactly what was responsible for the resistance observed in this study, since our analysis does not include determining the mechanism of the resistance. Nonetheless, previous studies have reported that widespread development of resistance against tetracycline is thought to be the result of pumping of the drug out of the extracellular space before it reaches its site of activity or due to changes in cellular permeability [35]. This is similar to an earlier report by Ngoi et al. in Malaysia, where 51% and 52% resistance was observed for tetracycline and streptomycin, respectively [36]; while a considerably low resistance was observed for CN, SXT, and S. This may imply that the drugs (gentamicin, sulfamethazine/trimethoprim, and streptomycin) may still have considerable activity against *Salmonella* as observed in the present study. On the other hand, the high level of resistance observed for tetracycline, ampicillin and streptomycin is in contrast to a similar study conducted in India, where the absence or low rate of resistance to ampicillin, and tetracycline was recorded [37]. Furthermore, relatively low resistance to CTX and other third-generation cephalosporins was found in the present study. This outcome confirms earlier reports from Malaysia and China [36,38].

The phenotypic and genotypic AMR profiling in the present study corresponds with the tetracycline resistance gene (*tetC*), the *aac* (6’) that confers resistance to aminoglycosides including gentamicin and the *bla*TEM β-lactam resistance gene. In each of the aforementioned resistance genes, isolates from cloacal swabs showed the highest amount of resistance followed by raw chicken meat and ready-to-eat meat. As earlier mentioned, *S*. Enteritidis is an enteric pathogen, which implies that animal faeces is always going to be an important source. Animals acquire infection by eating materials (e.g., feed, water, pasture grass) contaminated with faeces of other infected animals. Additionally, the level of *Salmonella* in livestock varies depending on the endemicity, production system and whether adequate control measures are in place. This result agrees with Kagambega et al. who detected a high prevalence of *S.* Enteritidis in the production of animals slaughtered for human consumption [39]. However, many discordant results were also recorded with the genetic evaluation proving to be superior and more robust compared to the phenotypic characterizations. While none of the isolates was positive for the *mcr* colistin resistance gene, the detection of the *Pmr_cef_* genes which are responsible for the mediation of resistance to colistin should be worrisome [26]. The *pmrA*-activated *pmrC* gene encodes an inner membrane protein that is required for the incorporation of phosphoethanolamine into lipid A and for polymyxin B resistance [40]. Colistin is a highly potent drug for the treatment of the majority of the multidrug-resistant Gram-negative bacteria [41]. It is considered the last-line choice of drug for the management of severe infection due to Gram-negative bacteria. Therefore, the emergence of resistance is viewed as a serious health problem. The presence of AMR genes *dfrA15*, *sul1*, *sul2*, *aadA1*, *aadA2*, *CpxR*, *floR* and *qnr* in these study isolates, have earlier been reported in *S*. Enteritidis [42,43].

*Salmonella* has been reported to acquire virulence from other species through horizontal gene transfer [44]. The acquisition of these virulence gene clusters is said to be the major driving force in the evolution and emergence of highly pathogenic strains [45,46]. Most of the virulence genes are located in a cluster referred to as the *Salmonella* Pathogenicity Island (SPI) within the chromosome [21]. In this study, all the isolates were found to possess SPI-1 that codes for the type III secretion system, which functions as the actin-binding proteins (SptP and SopE). This actin-binding protein is essential during the transportation and uptake of the bacterium by the cells. In addition, the 40 kb SPI-2 locus that encodes the second type III secretion system was also detected, and this is important for the survival of the bacteria in epithelial cells and macrophages [47,48]. In addition to the SPI 1 and SPI 2 locus, the *mgtC* gene and the plasmid-encoded fimbriae chaperone gene *PefD* were also detected in this study. These genes are responsible for the growth of the bacteria in an Mg^2+^-limiting environment such as phagosomes, and the adhesion of *S.* Enteritidis to the small intestine, respectively. This finding is in concordance with previous reports where they identified that the *Salmonella* pathogenicity islands are chromosomal clusters of horizontally acquired virulence genes [49,50,51]. Furthermore, 88% of the isolates were found to possess the plasmid virulence (*spv*) locus. Notably among these are *spvB* and *spvC*, which are responsible for the multiplication of intracellular *Salmonella* [52]. A similar finding has also been reported where experiments show that the simultaneous administration of *spvB* and *spvC* are capable of conferring sufficient virulence to *Salmonella* species in mice [53]. The detection of virulence genes within these pathogenicity islands implies that the *S. Enteritidis* isolates found in this study are highly virulent and poses a potential threat of worse disease outcome in susceptible humans and animals.

According to the MLST analysis, the *S*. Enteritidis study isolates were typed into seven genotypes, with the majority of the isolates clustering into ST1925 (63%) followed by ST11 (26.5%), with ST292 (raw chicken meat), ST329 (cloacal swabs), ST365 (read-to-eat chicken meat), ST423 (raw chicken meat) and ST2132 (cloacal swab) occurring singly in each case. Earlier reports indicate that among *S.* Enteritidis worldwide, ST11 is the predominant sequence type, accounting for 89% of the sequence types in the EnteroBase database [54]. Similarly, according to the goeBurst analysis, these sequence types share some genetic features by their close relationship. This observation is supported by an earlier report where ST1925 is said to be a variant of the ST11 sequence type [55]. The multilocus sequence types of *S*. Enteritidis previously reported in Malaysia include ST11 and ST1925, isolated from poultry [56]. However, in neighbouring Singapore, ST11 and ST1925 were reported from *Salmonella* isolated from retail food and wild birds [57]. Moreover, ST11 has been reported as one of the most common genotypes of *S*. Enteritidis in Queensland, Australia [58]. Other novel ST types detected in this study are ST292, ST329, ST423 and ST2132. The distribution of these rare sequence types may signify the role of human travel and trade in exotic animal species from endemic regions in the spread of AMR-resistant *Salmonella* subtypes, which could pose an important health hazard. Likewise, the detection of these novel STs constitute a serious public health challenge because earlier studies that detected *Salmonella* (*S.* Albany, *S.* Ohio, and *S.* Kentucky) harbouring resistance to cephalosporins and macrolide antibiotics were found to also belong to these sequence types [57,59]. In addition, the isolates showing resistance to multiple antimicrobials clustered in the novel ST2132 as well as the predominant ST11 sequence types. However, an endemic *Salmonella* genotype reported in Asia, ST34 [37], was not observed in this study. Each of the aforementioned sequence types has been repeatedly reported in food, poultry and human clinical samples from the United Kingdom and the United States [57,59,60].

## 5. Conclusions

The study provides an appraisal of the AMR, virulence determinants and the MLST subtypes of *S*. Enteritidis isolates circulating in Malaysia. It is important to highlight that although resistance to some of the antimicrobials tested was observed, none of the isolates was found to show resistance to colistin as no *mcr* gene was detected. We also noticed that the isolates possess a number of *Salmonella* virulence determinants which is an indication that they are highly pathogenic, hence possessing a serious threat to humans. Further, the in silico serotype prediction proved to be a good alternative to the traditional Kauffman–White Scheme for serotyping of *Salmonella* by providing good insights into the genetic determinants of *Salmonella*.

## 6. Study Limitation

This study was not able to discuss adequately the epidemiological aspect of the *S.* Enteritidis isolates obtained because we did not have full access to the sampling and sources of the samples.

## Figures and Tables

**Table 1:** *S.* Enteritidis isolates showing resistance to antimicrobial drugs tested.

Antimicrobials	Percentage of *Salmonella* Isolates			Total (*n* = 45)
	Ready-to-Eat Chicken Meat (*n* = 7)	Fresh Chicken Meat (*n* = 11)	Cloacal Swab (*n* = 27)	
Amp (10 µg)	-	-	6 (22%)	6 (13.3%)
C (30 µg)	-	1 (9.1%)	-	1 (2.2%)
CN (10 µg)	-	-	1 (3.7%)	1 (2.2%)
S (10 µg)	-	-	1 (3.7%)	1 (2.2%)
SXT (25 µg)	-	1 (9.1%)	-	1 (2.2%)
TE (30 µg)	5 (71.4%)	4 (36.4%)	11 (40.7%)	20 (44.4%)
EFT (30 µg)	-	-	-	-
CTX (30 µg)	-	-	-	-
CIP (5 µg)	-	-	-	-

Ampicillin (Amp), chloramphenicol (C), gentamicin (CN), streptomycin (S), sulfamethazine/trimethoprim (SXT), tetracycline (TE), ceftiofur (EFT), cefotaxime (CTX), and ciprofloxacin (CIP).

**Table 2:** In silico WGS and phenotypic *Salmonella* serotypes.

S/No.	WGS Accession No.	Sample Name	SeqSero Antigenic Formula/Serotype	Phenotypic Method
1	JAECPG000000000	S34	9:g,m:-/Predicted serotype: Enteritidis	Enteritidis
2	JAECPF000000000	S35	9:g,m:-/Predicted serotype: Enteritidis	Enteritidis
3	JAECPC000000000	S38	9:g,m:-/Predicted serotype: Enteritidis	Enteritidis
4	JAECPB000000000	S39	9:g,m:-/Predicted serotype: Enteritidis	Enteritidis
5	JAECPA000000000	S40	9:g,m:-/Predicted serotype: Enteritidis	Enteritidis
6	JAECOZ000000000	S41	9:g,m:-/Predicted serotype: Enteritidis	Enteritidis
7	JAECOY000000000	S42	9:g,m:-/Predicted serotype: Enteritidis	Enteritidis
8	JAECOX000000000	S43	9:g,m:-/Predicted serotype: Enteritidis	Enteritidis
9	JAECOW000000000	S44	9:g,m:-/Predicted serotype: Enteritidis	Enteritidis
10	JAECOV000000000	S45	9:g,m:-/Predicted serotype: Enteritidis	Enteritidis
11	JAECOU000000000	S46	9:g,m:-/Predicted serotype: Enteritidis	Enteritidis
12	JAECOT000000000	S47	9:g,m:-/Predicted serotype: Enteritidis	Enteritidis
13	JAECOS000000000	S48	9:g,m:-/Predicted serotype: Enteritidis	Enteritidis
14	JAECOR000000000	S49	9:g,m:-/Predicted serotype: Enteritidis	Enteritidis
15	JAECOQ000000000	S50	9:g,m:-/Predicted serotype: Enteritidis	Enteritidis
16	JAECOP000000000	S51	9:g,m:-/Predicted serotype: Enteritidis	Enteritidis
17	JAECOO000000000	S52	9:g,m:-/Predicted serotype: Enteritidis	Enteritidis
18	JAECON000000000	S53	9:g,m:-/Predicted serotype: Enteritidis	Enteritidis
19	JAECOM000000000	S54	9:g,m:-/Predicted serotype: Enteritidis	Enteritidis
20	JAECOL000000000	S56	9:g,m:-/Predicted serotype: Enteritidis	Enteritidis
21	JAECOK000000000	S57	9:g,m:-/Predicted serotype: Enteritidis	Enteritidis
22	JAECOJ000000000	S59	9:g,m:-/Predicted serotype: Enteritidis	Enteritidis
23	JAECOI000000000	S60	9:g,m:-/Predicted serotype: Enteritidis	Enteritidis
24	JAECOH000000000	S61	9:g,m:-/Predicted serotype: Enteritidis	Enteritidis
25	JAECOG000000000	S63	8:z4,z24:-/Predicted serotype: Albany/Duesseldorf	Enteritidis
26	JAECOF000000000	S64	9:g,m:-/Predicted serotype: Enteritidis	Enteritidis
27	JAECOE000000000	S65	9:g,m:-/Predicted serotype: Enteritidis	Enteritidis
28	JAECOD000000000	S66	9:g,m:-/Predicted serotype: Enteritidis	Enteritidis
29	JAECOC000000000	S67	9:g,m:-/Predicted serotype: Enteritidis	Enteritidis
30	JAECOB000000000	S68	9:g,m:-/Predicted serotype: Enteritidis	Enteritidis
31	JAECOA000000000	S69	9:g,m:-/Predicted serotype: Enteritidis	Enteritidis
32	JAECNZ000000000	S70	9:g,m:-/Predicted serotype: Enteritidis	Enteritidis
33	JAECNY000000000	S71	9:g,m:-/Predicted serotype: Enteritidis	Enteritidis
34	JAECNX000000000	S72	7:b:l,w/Predicted serotype: Ohio	Enteritidis
35	JAECNW000000000	S73	9:g,m:-/Predicted serotype: Enteritidis	Enteritidis
36	JAECNV000000000	S74	9:g,m:-/Predicted serotype: Enteritidis	Enteritidis
37	JAECNU000000000	S75	9:g,m:-/Predicted serotype: Enteritidis	Enteritidis
38	JAECNT000000000	S76	9:g,m:-/Predicted serotype: Enteritidis	Enteritidis
39	JAECNS000000000	S77	3,10:r:z6/Predicted serotype: Weltevreden	Enteritidis
40	JAECNR000000000	S78	9:g,m:-/Predicted serotype: Enteritidis	Enteritidis
41	JAECNQ000000000	S79	9:g,m:-/Predicted serotype: Enteritidis	Enteritidis
42	JAECNP000000000	S80	9:g,m:-/Predicted serotype: Enteritidis	Enteritidis
43	JAECNO000000000	S81	8:i:z6/Predicted serotype: Kentucky	Enteritidis
44	JAECOV000000000	S45	9:g,m:-/Predicted serotype: Enteritidis	Enteritidis
45	JAECNN000000000	S82	9:g,m:-/Predicted serotype: Enteritidis	Enteritidis

**Table 3:** Distribution of antimicrobial resistance AMR genes in *Salmonella* Enteritidis isolates based on the antimicrobial susceptibility test AST.

Gene	*Aac (6′)-Iy*	*Pmr c*	*Pmr e*	*Pmr f*	*Bla*_TEM-4_	*Bla*_TEM-33_	*Dfra15*	*TetC*	*Sul 1*	*Sul 2*	*flor*	*Qnr*
Ready-to-eat chicken meat (*n* = 7)	7	7	7	7	-	-	-	5	-	-	-	-
Fresh chicken meat (*n* = 11)	10	7	7	7	1	1	2	8	1	1	1	1
Cloacal swab samples (*n* = 27)	24	25	25	25	6	6	-	6	-	-	-	-

**Table 4:** MLST sequence types of *Salmonella* Enteritidis isolates.

Sequence Types	ST11	ST292	ST329	ST365	ST423	ST1925	ST2132
Ready-to-eat chicken meat	2	NT	NT	1	NT	5	NT
Fresh chicken meat	2	1	NT	NT	1	12	NT
Cloacal swabs	9	NT	1	NT	NT	14	1

NT—Not Type-able.

## Data Availability

Publicly available datasets were analyzed in this study. This data can be found here. (https://www.ncbi.nlm.nih.gov/biosample?Db=biosample&DbFrom=bioproject&Cmd=Link&LinkName=bioproject_biosample&LinkReadableName=BioSample&ordinalpos=1&IdsFromResult=674970 (accessed on 26 October 2021)).
